# A Randomised Controlled Trial of Clinician-Guided Internet-Based Cognitive Behavioural Therapy for Depressed Patients in Singapore

**DOI:** 10.3389/fpsyg.2021.668384

**Published:** 2021-07-29

**Authors:** Sharon H. X. Lu, Hanita A. Assudani, Tammie R. R. Kwek, Shaun W. H. Ng, Trisha E. L. Teoh, Geoffrey C. Y. Tan

**Affiliations:** ^1^Department of Psychology, Institute of Mental Health, Singapore, Singapore; ^2^Department of Mood and Anxiety, Institute of Mental Health, Singapore, Singapore; ^3^Singapore Institute of Clinical Sciences, Agency for Science Technology and Research (A*STAR), Singapore, Singapore; ^4^Clinical Imaging Research Centre, Yong Loo Lin Medical School, National University of Singapore, Singapore, Singapore

**Keywords:** internet treatments, cognitive behavioural therapy, depression, Asians, randomised control trial

## Abstract

This study examined the efficacy and acceptability of a hybrid, clinician-guided internet-based Cognitive Behavioural Therapy (iCBT) programme for outpatients with depression in a psychiatric hospital in Singapore. Fifty three participants were randomly assigned to a treatment or wait-list control group before they underwent a cross-over of conditions. Treatment consisted of a 4-week iCBT with three face-to-face sessions. 60.9% of participants who received treatment completed all six modules. Intention-to-treat analysis showed treatment was associated with significant reductions in symptoms of depression, anxiety and psychological distress but not in functional impairment, while the control condition was not associated with changes in any measures. These reductions had moderate to large effect sizes (ESs) for symptoms of depression and anxiety, and moderate ES for psychological distress. The between-group difference in depression score had a moderate ES. There was a significant between-group treatment effect in depressive symptoms, but not in the other measures. Treatment gains were maintained at 3-month follow-up. Most of the participants were highly satisfied with the programme, with 90 percent stating they would recommend it. This is the first RCT to provide preliminary evidence for the efficacy and acceptability of iCBT for depression in Singapore.

## Introduction

Depression is the leading cause of disability globally (Kessler et al., 2003, 2006). Depression is highly comorbid with other mental and physical conditions and its presence exacerbates dysfunction, and burden on health services (Angst et al., 1999). It is estimated that 6.3% of Singaporeans will have depression and 1.6% will have anxiety at some point, with 3.5% suffering from at least two mental health disorders in their life (Subramaniam et al., 2020).

Cognitive Behavioural Therapy (CBT) is a mainstay in evidence-based treatments for depression and anxiety. However, 59.6% of Singaporeans with depression do not access professional help (Chong et al., 2012). Previous studies have shown that the barriers to accessing treatment in Asian populations include stigma, concerns about treatment costs, transportation difficulties and a shortage of culturally appropriate services (Lu et al., 2013). Furthermore, access to psychotherapy may also be limited during periods of emergency, as witnessed during the ongoing COVID-19 pandemic where multiple countries have gone into lockdown and healthcare services have been restricted (Yang et al., 2020).

One strategy that has been shown to increase access to treatments is internet-delivered CBT (iCBT) (Abbott et al., 2008; Barak et al., 2009; Ryan et al., 2010). iCBT involves the delivery of skills and information based on the CBT approach, *via* the internet. Clinician guidance in the form of telephone and/or email coaching is often provided concurrently and has been shown to improve effect sizes and adherence (Andersson and Cuijpers, 2009; Talbot, 2012). iCBT is a cost-effective and efficacious treatment for depression and anxiety as singular disorders (Spek et al., 2007; Andrews et al., 2010) or for comorbid depression and anxiety (Titov et al., 2010, 2011, 2012; Dear et al., 2011; Johnston et al., 2011).

While iCBT has shown efficacy in Western populations, limited evidence exists for Asian populations. Ooi et al. (2016) reported web-based CBT programmes to be efficacious in decreasing the severity of selective mutism among affected Singaporean children. Choi et al. (2012) reported a culturally adapted version of iCBT to be efficacious in treating depression among Chinese-speaking immigrants in Australia. However, to our knowledge, no studies have yet examined the efficacy of iCBT for treating depression in an Asian setting.

As such, the present study would be the first of its kind to explore the feasibility of delivering iCBT to depressed patients in Singapore. Given that the introduction of delivering CBT *via* the internet is relatively new to the population in Singapore, the authors aimed to scaffold the delivery of iCBT with face-to-face support provided by a clinician, instead of relying solely on email or phone support. Feedback from users on the acceptability of a hybrid model of iCBT as a psychotherapeutic treatment option will also be obtained.

The present study aims to explore the efficacy and acceptability of a blended iCBT programme, with three face-to-face sessions conducted by a clinician, for patients with depression in Singapore.

## Method

### Design, Sample Size, and Hypotheses

**Figure 1:**
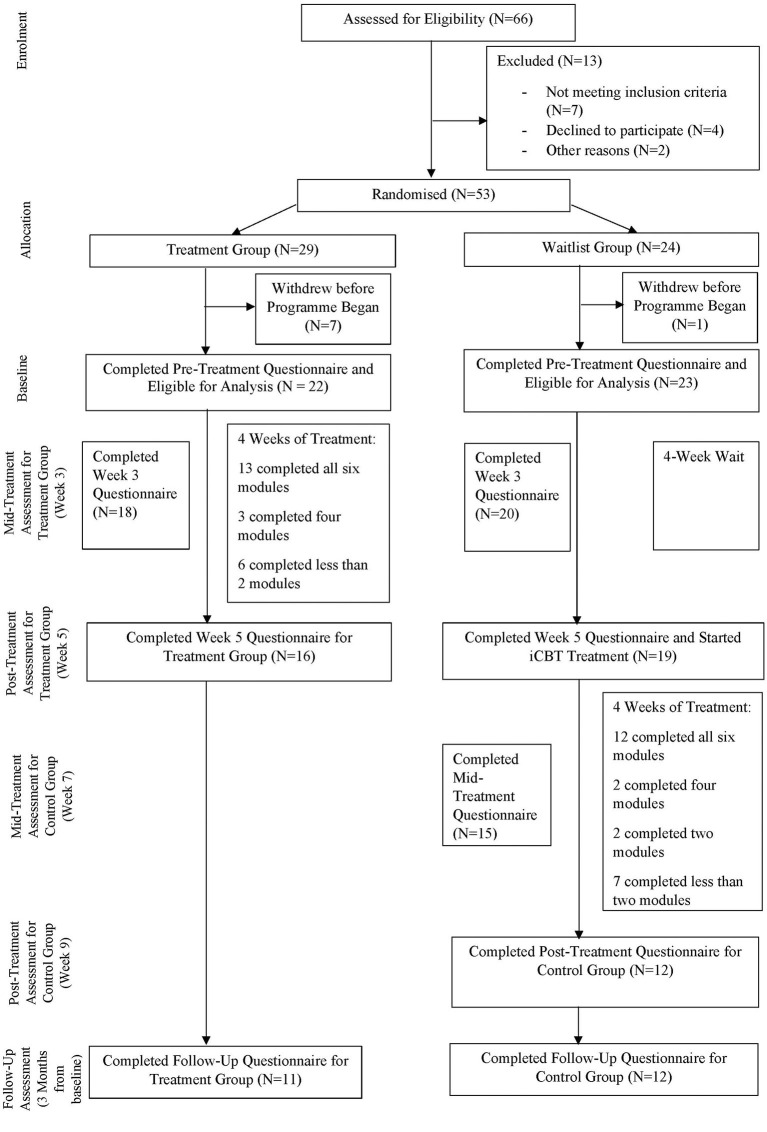
Participants flow chat.

The current study involves a cross-over, randomised controlled trial between a treatment group and a delayed waitlist control group. The control group underwent the same intervention with clinician support after waiting 4 weeks (Figure 1).

Power calculations for a cross-over randomised controlled trial determined that a total sample size of 32 was sufficient to detect an effect size of 0.5 (power at 80% and alpha at 0.05), which is the minimum expected based on a similar study (Choi et al., 2012).

It was hypothesised that compared to control, treatment would be associated with greater pre-post improvements in depressive symptoms. These gains would also be sustained at 3-month follow-up. Lastly, participants would find iCBT to be acceptable.

### Participants and Recruitment

Outpatients from the Institute of Mental Health who presented with mild to moderate depressive symptoms were invited to participate in the current study from 2017 to 2018. The study had to be terminated prematurely due to the unexpected closure of the server which hosted the online programme, even though the recruitment target has not been met. The inclusion criteria were: (1) aged 21 to 65 yr old, (2) not currently seeing a therapist for any form of individual or group therapy, (3) presented with mild to moderate depressive symptoms, (4) if on medication for depression, to be on a stable dose for at least a month, (5) provided informed consent, (6) able to read and speak at least Primary 6 level English, and (7) adequate computer skills to operate the iCBT programme. All participants recruited had undergone formal psychiatric assessment with a psychiatrist in the hospital and presented with primarily depressive symptoms.

The exclusion criteria were: (1) severe depression (i.e., score ≥ 23 on PHQ-9), (2) strong suicidal ideation (i.e., score > 2 on Question 9 of the PHQ-9), (3) current active suicidal intention or plan, (4) depression was not the presenting problem, (5) did not meet the minimum English language requirements, and (6) concurrently attending other forms of group/individual psychotherapy.

The study was approved by the ethics board, the National Healthcare Group Domain Specific Review Board (DSRB) in Singapore (Protocol Record 2015/00404) and registered with ClinicalTrials.gov as NCT04100785.

### Primary Outcome Measure

#### Patient Health Questionnaire-9 (PHQ-9)

The PHQ-9 (Kroenke et al., 2001) is a nine-item self-report measure of symptoms and severity of major depressive disorder, based on the Diagnostic and Statistical Manual of Mental Disorders, Fourth Edition (DSM-IV; American Psychiatric Association, 2000) criteria for Major Depressive Disorder. Scores on each item range from 0 (not at all) to 3 (nearly every day). An overall cut-off score of 10 or greater is sensitive to a DSM-IV diagnosis of depression (Kroenke et al., 2010). The PHQ-9 has an internal reliability of 0.86–0.89, a sensitivity of 88%, and a specificity of 88% for the clinical cut-off of 10. It also has an excellent test-retest reliability of 0.84 and correlates moderately (*r* = 0.58) with the SF-20 mental health scale (Kroenke et al., 2001). The PHQ-9 has also been found to be valid and reliable for screening depression in Singapore (Sung et al., 2013).

### Secondary Outcome Measures

#### Generalised Anxiety Disorder-7 (GAD-7)

The GAD-7 (Spitzer et al., 2006) is a seven-item self-report measure of symptoms and severity of Generalised Anxiety Disorder (GAD), based on the DSM-IV criteria for GAD. Scores for each item range from 0 (not at all) to 3 (nearly every day). An overall cut-off score of 10 or greater is sensitive to DSM-IV diagnoses of GAD, social phobia, and panic disorders (Kroenke et al., 2007). The GAD-7 has an internal reliability of 0.92, test-retest reliability of 0.83, and criterion validity of 0.75 with the SF-20 mental health scale (Spitzer et al., 2006). The GAD-7 is increasingly adopted in empirical studies and large-scale dissemination studies as a general measure of shifts in anxiety symptoms (Clark et al., 2009).

#### Kessler-10 Item (K-10)

The K-10 (Kessler et al., 2002) is a ten-item self-report measure of non-specific psychological distress and strong support has been found between the K-10 and its associations with diagnoses of anxiety and depression (Andrews and Slade, 2001). Scores for each item ranged from 0 (no distress) to 5 (highly distressed), with total scores spanning from 0 to 50. The scale possesses excellent internal consistency (α = 0.93), even with ethnically diverse populations (Fassaert et al., 2009).

#### Work and Social Adjustment Scale (WSAS)

The WSAS (Mundt et al., 2002) is a five-item self-report measure of the extent of impairment in work and social functioning, with scores ranging from 0 to 40. An overall score of above 20 appears to suggest moderately severe or worse psychopathology. Scores between 10 and 20 are associated with significant functional impairment but less severe clinical symptomatology. The WSAS has an internal reliability of 0.70–0.94 and a test-retest reliability of 0.73. Its convergent validity compared with clinical interviews and severity of depression is 0.76–0.86.

#### Timepoints

Participants in the treatment group started the intervention after the first face-to-face meeting with a psychologist. On the other hand, participants in the delayed waitlist-control group received the intervention after 4 weeks.

The iCBT programme spanned over 4 weeks. The outcome measures were administered during the face-to-face session at Week 1 (before Module 1), Week 3 (mid-treatment), and Week 4 (after Module 6), as well as at three-month follow-up *via* email.

### Intervention

The iCBT programme consisted of six online modules adapted from the THRIVE programme, which is a community mental health self-help programme locally developed by Khoo Teck Puat Hospital in Singapore. The outline for each module is as follows: (1) introduction to depression, (2) problem-solving, (3) understanding the influence of thoughts and beliefs in depression, (4) overcoming negative thoughts and beliefs, (5) planning meaningful activities (part 1), and (6) planning meaningful activities (part 2). Participants were instructed to complete Modules 1 and 2 in week 1; Modules 3 and 4 in week 2 and week 3; Modules 5 and 6 in week 4.

In addition to the online modules, there were three face-to-face 30-min sessions with a psychologist conducted at weeks 1, 3, and 4. The face-to-face sessions were recommended by the ethics board to ensure that patients presenting to an acute psychiatric hospital were able to receive a minimal standard of care—one component being face-to-face contact with a clinician. These sessions were for patients to discuss the application of the content of the modules in relation to their presenting problems. It should be noted that these face-to-face sessions were not therapy sessions, and the role of the psychologists in the present study was solely to provide support and encouragement, as well as to assist the participants in applying the content of the modules. These sessions were arranged to maintain contact so as to promote adherence to the online treatment and reduce dropout rates. Moreover, administrative matters could also be settled, such as resolving technical issues, completing questionnaires, and obtaining ongoing feedback.

### Therapists

Two clinical psychologists with postgraduate training in clinical psychology provided all face-to-face contact (three sessions) with participants. The content of the face-to-face contact followed a specific structure, where opportunities for reinforcing participants, guidelines for answering frequently asked questions, and additional information were provided.

### Procedure

Upon expressing their interest in the study, participants were contacted by the researchers to arrange for a first face-to-face meeting. During this meeting, the researchers explained the purpose of the study, the procedures involved, obtained informed consent from the participants to take part in the study and administered the baseline questionnaires. Participants were then randomised to either the intervention or the delayed waitlist control group *via* a random number generator. After being assigned to a group, participants were briefed on the procedures depending on the group they belonged to. Participants in the treatment group were also briefed on log-in details and given information about assessing the iCBT materials.

Following the first face-to-face meeting, participants from the treatment group underwent 1 week of online modules, followed by the second face-to-face meeting, where they completed the mid-treatment measures, followed by another week of online modules before the third face-to-face meeting, where the post-treatment measures were taken. Similarly, participants from the control group completed the mid-treatment and post-treatment measures on the third and fifth week *via* email following the first face-to-face meeting. After the completion of the post-treatment measures, participants from the delayed waitlist control group commenced their treatment within the same week. At the end of the study, participants were remunerated with a small inconvenience fee for their participation.

### Analyses

The study employed a cross-over randomised design where each group underwent an initial comparison arm, followed by a cross-over arm. In the initial comparison arm, the group undergoing treatment initially was referred to as the “treatment group,” while the waitlist group was designated as the “control group.” Differences between treatment and control groups in demographic data and pre-treatment scores were analysed using two-sample *t*-tests and chi-square tests. Baseline differences between completers and non-completers were analysed using two-sample *t*-tests. In line with previous RCTs, univariate ANCOVAs were conducted to compare treatment and control groups in the initial comparison arm, analysing for differences at week 5 while controlling for baseline scores (Choi et al., 2012). Repeated measures General Linear Models (rmGLM) were used to analyse data collected at pre-treatment, post-treatment, post-cross-over, and follow-up. To reduce potential biases from attrition, rmGLM were used for longitudinal analysis of the cross-over design across both arms and per-protocol and intention-to-treat analyses were performed. Effect sizes (ESs; Cohen's d) were calculated to quantify the magnitude of change in symptoms for within and between groups, based on the pooled standard deviation. All analyses were performed in PASW version 25.0 (SPSS Inc., Chicago, IL).

Our primary outcome measure was the PHQ-9 as the intervention was designed to target depressive symptoms. Secondary outcome measures comprised the GAD-7, WSAS, and K-10. Regarding the cross-over design, there are two dependent variables: (i) control response, which is the response under the control condition, and (ii) treatment response, which is the response under the iCBT condition and both variables are deviations from each subject's baseline measurements including order of intervention as a between-subject factor. Longitudinal effects of treatment and control were further analysed in each arm. We conducted both per-protocol and intention-to-treat analyses.

Clinical significance was examined using remission and recovery rates. Remission is defined as the proportion of participants who initially scored at or above, but subsequently scored below the cut-off scores of 10 for the PHQ-9 total score (Kroenke et al., 2001, 2010; Gilbody et al., 2007) and 8 for the GAD-7 total score. Secondly, clinical recovery is estimated using the proportion of participants who showed a significant reduction of 50 percent of pre-treatment PHQ-9 and GAD-7 scores, as described in previous studies (Richards and Suckling, 2009; Titov et al., 2011).

Thematic analysis of the feedback obtained from open-ended questions (“Please elaborate on what you have found to be helpful and not helpful in this programme”; “Is there anything we can do to make the iCBT programme more useful?”) was conducted to generate a list of preliminary themes that participants found useful about iCBT in this current study.

## Results

### Demographic Characteristics of Participants

The mean age of participants was 33.1 years (*SD* = 10.5), and 24 (53.3%) of them were males. Other relevant demographic characteristics of the participants are included in Table 1. Chi-squared tests revealed no significant differences between groups on all the demographic characteristics of the participants (*p* > 0.05). There was no significant difference in pre-treatment PHQ-9 scores between the treatment group and the control group, *t*_(44)_ = 0.08, *p* = 0.94.

**Table 1:** Demographic characteristics of the participants.

**Variable**	**Treatment group**		**Control group**		**Total**		**Significance statistics**
	***n***	**%**	***n***	**%**	***n***	**%**	
Gender							
Male	11	50	13	56.5	24	53.3	*x*^2^ = 0.19, *p* = 0.66
Female	11	50	10	43.5	21	46.7	
Age							
Mean	36.1 (12.1)		30.3 (8.0)		33.1 (10.5)		*t*(43) = 1.92, *p* = 0.06
Range	21 to 58		20 to 48		20 to 58		
Marital status							
Single	12	54.5	17	73.9	29	64.4	*x*^2^ = 1.98, *p* = 0.37
Married	6	27.3	3	13.0	9	20.0	
Divorced/widowed	4	18.2	3	13.0	7	15.6	
Highest educational level							
Secondary School	4	18.2	2	8.7	6	13.3	*x*^2^ = 0.90, *p* = 0.64
Diploma/junior college	9	40.9	11	47.8	20	44.4	
Graduate/ post graduate	9	40.9	10	43.5	19	42.2	
Employment status							
Student	3	13.6	1	4.3	4	8.9	*x*^2^ = 2.19, *p* = 0.335
Employed	13	59.1	18	78.3	32	68.9	
Unemployed	6	27.3	4	17.4	10	22.2	
Taking medication							
No	8	36.4	7	30.4	17	33.3	*x*^2^ = 1.27, *p* = 0.529
Depression	9	40.9	13	56.5	22	48.9	
Depression and Anxiety	5	22.7	3	13.0	8	17.8	
Previously had therapy							
Yes	15	68.2	13	56.5	28	62.2	*x*^2^ = 0.65, *p* = 0.420
No	7	31.8	10	43.5	17	37.8	
Previously done CBT							
Yes	6	27.3	4	17.4	10	22.2	*x*^2^ = 0.64, *p* = 0.425
No	16	72.7	19	82.6	35	77.8	
Internet use per week							
< 10 h	5	22.7	3	13.0	8	17.8	*x*^2^ = 0.81, *p* = 0.668
10 to 35 h	11	50.0	12	52.2	23	51.1	
More than 35 h	6	27.3	8	34.8	14	31.1	

### Adherence and Attrition

Out of the 29 participants who were assigned to the treatment group, seven (24.1%) did not attend the first face-to-face session. 22 people completed the pre-treatment questionnaires and were eligible for analysis. Out of the 22 participants in the treatment group who started treatment, 13 (59.1%) completed all six modules within the 4 weeks of the intervention, three (13.6%) completed four modules, and six (27.3%) completed less than two modules. The average number of complete modules was 4.09 (*SD* = 2.65). 11 (50%) completed the three-month follow-up questionnaire.

Out of the 24 participants who were in the control group, one (4.2%) did not complete the pre-treatment questionnaire. 23 people (95.8%) completed at least the pre-treatment questionnaire and were included in the analyses. Out of the 23 participants, 19 started the iCBT intervention at Week 5.12 (63.2%) completed all six modules, two (10.5%) completed four modules, two (10.5%) completed two modules, and seven (36.8%) completed less than two modules. In total, 25 out of 41 (60.9%) of participants who received the intervention completed all six modules. The average number of completed modules was 3.73 (*SD* = 2.78). 12 (52.2%) completed the post-treatment questionnaire and 12 (52.2%) completed the three-month follow-up questionnaire.

Pre-treatment symptom scores were analysed using independent 2-sample *t*-tests to compare those who completed the post-treatment questionnaire and those who did not. From a total of 45 participants, there were 35 completers and 10 non-completers. As compared to non-completers, completers did not have significant differences in their baseline scores for PHQ-9 (mean difference = 1.93, *SE* = 1.94, *t* = 0.992, *p* = 0.33), GAD-7 (mean difference = 1.69, *SE* = 2.15, *t* = 0.78, *p* = 0.44), WSAS (mean difference 6.50, *SE* = 3.23, *t* = 2.01, *p* = 0.051) and K10 (mean difference = 5.25, *SE* = 3.15, *t* = 1.67, *p* = 0.10) .

### Treatment Outcomes

The initial comparison arm was analysed for between-group differences in post-treatment scores, controlling for pretreatment scores, between the treatment group and the control group.

#### Treatment vs. Control in the Initial Comparison Arm

In the per-protocol analyses, the between-group difference in post-treatment PHQ-9 scores was significant in the initial comparison arm, (*F*_1, 34_ = 4.96, *p* = 0.033). The between-group difference was not significant for the GAD-7 (*F*_1, 34_ = 2.45, *p* = 0.13), WSAS (*F*_1, 34_ = 1.54, *p* = 0.22), and K-10 (*F*_1, 34_ = 0.68, *p* = 0.42).

In the intention-to-treat analyses of the between-group difference in post-treatment scores there were no significant differences for the PHQ-9**, (***F*_1, 44_ = 1.29, *p* = 0.26), GAD-7, (*F*_1, 44_ = 0.52, *p* = 0.47), WSAS (*F*_1, 44_ = 0.96, *p* = 0.33) and K-10 (*F*_1, 44_ = 0.073, *p* = 0.79).

#### Treatment Response Across Both Arms

Treatment response across initial comparison and cross-over arms was analysed with rmGLMs from pre-treatment to post-treatment for both arms, with order as a between-subject factor.

Per-protocol analysis revealed a significant treatment response for the PHQ-9 (*F*_1, 26_ = 9.96, *p* = 0.004) and GAD-7 (*F*_1, 26_ = 11.54, *p* = 0.002). There was no significant treatment response observed for the WSAS (*F*_1, 26_ = 1.99, *p* = 0.171). There was a marginally significant treatment response observed for the K-10 (*F*_1, 26_ = 3.86, *p* = 0.060).

Intention-to-treat analyses revealed a significant treatment response for the PHQ-9 was significant (*F*_1, 43_ = 7.06, *p* = 0.011), GAD-7 (*F*_1, 43_ = 7.19, *p* = 0.010) and K-10 (*F*_1, 43_ = 4.71, *p* = 0.036). The treatment response for the WSAS was marginally significant (*F*_1, 43_ = 3.84, *p* = 0.057).

#### Control Response Across Both Arms

Control response across initial comparison and cross-over arms was analysed with rmGLMs from pre-control to post-control for both arms, with order as a between-subject factor.

In the per-protocol analyses, there were no significant control responses for the PHQ-9 (*F*_1, 25_ = 0.44, *p* = 0.51), GAD-7 (*F*_1, 25_ = 0.36, *p* = 0.55), WSAS (*F*_1, 25_ = 0.0004, *p* = 0.98) and K-10 (*F*_1, 25_ = 0.11, *p* = 0.74).

In the intention-to-treat analyses, there were no significant control responses for the PHQ-9 (*F*_1, 43_ = 1.54, *p* = 0.22), GAD-7 (*F*_1, 43_ = 0.35, *p* = 0.56), WSAS (*F*_1, 43_ = 0.0004, *p* = 0.99) and K-10 (*F*_1, 43_ = 0.76, *p* = 0.39).

#### Comparison Between Treatment and Control Responses in Both Arms

In the per-protocol analyses, there were no significant differences in the within-individual comparison between treatment and control responses in the PHQ-9 (*F*_1, 21_ = 0.63, *p* = 0.44), GAD-7 (*F*_1, 21_ = 1.98, *p* = 0.17), WSAS (*F*_1, 21_ = 0.037, *p* = 0.85) and K-10 (*F*_1, 21_ = 0.069, *p* = 0.80).

In the intention-to-treat analyses, there were no significant differences in the within-individual comparison between treatment and control responses in the PHQ-9 (*F*_1, 43_ = 1.59, *p* = 0.21), GAD-7 (*F*_1, 43_ = 1.90, *p* = 0.18), WSAS (*F*_1, 43_ = 1.76, *p* = 0.19), and K-10 (*F*_1, 43_ = 0.87, *p* = 0.36).

#### Follow-Up After Treatment Across Both Arms

The follow-up time-point was analysed by rmGLMs to determine whether treatment gains were maintained from baseline to follow-up and whether there were any changes from the post-intervention time point (week 5 for the initial treatment group and week 9 for the delayed wait-list group) to the 3-month follow-up timepoint.

In the per-protocol analyses, treatment gains were maintained from baseline to follow-up on the PHQ-9 (*F*_1, 21_ = 13.2, *p* =0.002), GAD-7 (*F*_1, 21_ = 8.14, *p* = 0.010) and K-10 (*F*_1, 21_ = 5.20, *p* = 0.033), while there was no significant change from baseline to follow-up in the WSAS (*F*_1, 21_ = 2.69, *p* = 0.12). This was supported by the lack of any significant changes between post-treatment and follow-up on the PHQ-9 (*F*_1, 10_ = 0.74, *p* =0.41), GAD-7(*F*_1, 10_ = 0.011, *p* = 0.92), WSAS (*F*_1, 10_ = 0.035, *p* = 0.86) and K-10 (*F*_1, 10_ = 0.12, *p* = 0.73).

In the intention-to-treat analyses, treatment gains were maintained from baseline to follow-up on the PHQ-9 (*F*_1, 43_ = 9.71, *p* = 0.003), GAD-7 (*F*_1, 43_ = 7.71, *p* =0.008), and K-10 (*F*_1, 43_ = 4.67, *p* = 0.036), while there was a marginally significant change from baseline to follow-up in the WSAS (*F*_1, 43_ = 3.82, *p* = 0.057). This was supported by the lack of any significant changes between post-treatment and follow-up on the PHQ-9 (*F*_1, 43_ = 1.38, *p* =0.25), GAD-7 (*F*_1, 43_ = 1.76, *p* = 0.19), WSAS (*F*_1, 43_ = 0.38, *p* = 0.54) and K-10 (*F*_1, 43_ = 0.41, *p* = 0.52).

### Effect Size

The ESs of the intervention in the treatment group on the change in PHQ-9 scores from baseline to week 5 and follow-up were 0.72 and 1.05, respectively (Table 2). For the wait-list control group, the ES of the change from baseline to week 5 was 0.14. The between-group effect size at post-treatment was 0.78. After the control group received the intervention, the ES post-intervention was 0.59. Across both groups, the intervention had an ES of 0.51 as compared to 0.08 for non-intervention periods. The response to treatment as compared to the response to non-intervention had an ES of 0.51.

**Table 2:** Observed and estimated means, standard deviations, ESs (Cohen's d) for outcome measures.

	**Observed means**			**ESs**		**Treatment vs. control ESs**
	**Pre**	**Post**	**Follow-up** ** (3 months from week 1)**	**Pre to post**	**Pre to follow-up**	**Pre to post**
**PHQ-9**						
Treatment	11.95 (6.03)	7.63 (6.05)	6.73 (3.55)	0.72^*^	1.30^*^	0.78^*^
Control	12.78 (6.76)	11.84 (6.30)	NA	0.14	NA	NA
Control (cross-over)	11.84 (6.30)	7.83 (7.27)	6.83 (7.42)	0.59	0.73	NA
Treatment response	12.20 (5.66)	7.71 (6.47)	NA	0.74^*^	0.95^*^	0.51
Control response	10.62 (6.33)	9.97 (5.94)	NA	0.11	NA	NA
**GAD-7**						
Treatment	8.50 (5.20)	5.31 (4.53)	4.27 (4.05)	0.65	0.91^*^	0.27
Control Control (cross-over)	11.47 (6.74) 10.53 (5.76)	10.53 (5.76) 5.83 (5.02)	NA 5.78 (4.47)	0.15 0.87^*^	NA 1.05^*^	NA NA
Treatment response Control response	9.44 (5.49) 8.56 (6.36)	5.54 (4.66) 8.23 (5.97)	NA NA	0.77^*^ 0.05	0.97^*^ NA	0.33 NA
**WSAS**						
Treatment	15.32 (10.20)	9.31 (8.75)	9.00 (8.81)	0.63	0.66	0.36
Control Control (cross-over)	17.37 (8.91) 17.15 (10.26)	17.15 (10.26) 13.42 (10.24)	NA 12.38 (11.78)	0.02 0.37	NA 0.54	NA NA
Treatment Response Control Response	16.17 (10.14) 13.69 (9.31)	11.07 (9.46) 14.17 (10.40)	NANA	0.52 −0.05	0.59 NA	0.23 NA
**K10**						
Treatment	23.91 (8.04)	19.44 (8.86)	18.27 (6.48)	0.53	0.77^*^	0.66
Control Control (cross-over)	27.37 (9.96) 25.68 (10.03)	25.68 (10.03) 20.80 (11.24)	NA16.80 (11.22)	0.17 0.46^*^	NA0.73^*^	NA NA
Treatment Response Control Response	24.73 (8.95) 24.15 (10.03)	20.10 (9.93) 23.96 (9.42)	NA NA	0.49^*^ 0.02	0.74^*^ NA	0.30 NA

*Standard deviations are shown in parentheses. Pre, Pre-treatment; Post, Post-treatment; Follow-up, Three-month follow-up; PHQ-9, Patient Health Questionnaire 9-item; GAD-7, Generalised Anxiety Disorder 7-item*.

* *indicates statistically significant results*.

The ESs of the intervention in the treatment group on the change in GAD-7 scores from baseline to week 5 and follow-up were 0.65 and 0.91, respectively. For the wait-list control group, the ES of the change from baseline to week 5 was 0.15. The between-group ES at week 5 is 0.27. After the control group received the intervention, the ES post-intervention was 0.87. Across both groups, the intervention had an ES of 0.50 as compared to 0.04 for non-intervention periods. The response to treatment as compared to the response to non-intervention had an effect size of 0.33.

The ESs of the intervention in the treatment group on the change in WSAS scores from baseline to week 5 and follow-up were 0.63 and 0.66, respectively. For the wait-list control group, the ES of the change from baseline to week 5 was 0.02. The between-group ES at post-treatment is 0.36. After the control group received the intervention, the effect at post-intervention was 0.37. Across both groups, the intervention had an ES of 0.52 as compared to−0.05 for non-intervention periods. The response to treatment as compared to the response to non-intervention had an ES of 0.23.

The ESs of the intervention in the treatment group on the change in K-10 scores from baseline to week 5 and follow-up were 0.53 and 0.77, respectively. For the wait-list control group, the ES of the change from baseline to week 5 was 0.17. The between-group ES at post-treatment is 0.66. After the control group received the intervention, the effect at post-intervention was 0.46. Across both groups, the intervention had an ES of 0.49 as compared to 0.02 for non-intervention periods. The response to treatment as compared to the response to non-intervention had an ES of 0.30.

### Proportion Showing Clinically Significant Change

Chi-squared analyses indicated significant differences in remission between treatment and control groups at week 5 on the PHQ-9 (Chi-squared = 4.38, *p* = 0.036) and GAD-7 (Chi-squared = 8.01, *p* = 0.0046) (Table 3). However, significant differences were not observed in recovery at post-treatment on the PHQ-9 (Chi-squared = 2.02, *p* = 0.16) and GAD-7 (Chi-squared = 3.13, *p* = 0.08).

**Table 3:** Proportion of participants above and below cut-off scores of clinical significance (remission) and proportion demonstrating at least 50% reduction in pre-treatment scores (recovery).

	**Treatment group**		**Control group**		**Control group (cross-over)**	
**Measure**	**Proportion**	**%**	**Proportion**	**%**	**Proportion**	**%**
	**(Number of cases/total)**					
**PHQ-9**						
Baseline not meeting clinical significance (score < 10)	8/22	36.4	7/23	30.4		
Post-treatment score ≥ 10 (remission)	10/16	62.5	7/19	26.8	8/12	66.6
Post-treatment score ≤ 50% pre-treatment score (Recovery)	7/16	43.8	4/19	21.1	6/12	50
Follow-up score ≥ 10 (Remission)	8/11	72.7	NA	NA	10/12	83.3
Follow-up score ≤ 50% pre-treatment score (Recovery)	6/11	54.5	NA	NA	7/12	58.3
**GAD-7**						
Baseline score < 8	10/22	45.5	9/23	39.1		
Post-treatment score < 8 (Remission)	12/16	75	5/19	26.3	9/12	75
Post-treatment score ≤ 50% pre-treatment score (Recovery)	8/16	50	4/19	21.1	7/12	58.3
Follow-up score < 8 (Remission)	9/11	81.8	NA	NA	9/12	75
Follow-up score ≤ 50% pre-treatment score (Recovery)	5/11	45.5	NA	NA	7/12	58.3

### Therapist Time

The mean total time spent per treatment group participant was 176.32 min (*SD* = 49.62) and this included the telephone screening interview, sending of encouragement emails as well as face-to-face sessions. The total time spent for the control group participants included the telephone screening interview, sending of reminder emails to complete questionnaires, as well as telephone calls to remind participants to complete questionnaires if they did not complete said questionnaires post-email reminders The mean total time spent per Control group was 26.15 min (*SD* = 3.70).

### Treatment Satisfaction

The treatment group participants who completed the post-treatment feedback questionnaire reported a good level of satisfaction with the programme (refer to Table 4). When they were asked to provide a rating from 1 to 5, where 5 indicates the highest level of agreement. The treatment group participants rated that they were satisfied with the programme (*M* = 4.0, *SD* = 0.74), that the programme had helped with their low mood (M = 3.58, *SD* = 0.79), and that they were likely to use the skills that they learnt from this programme if they were to encounter depressive symptoms again (M = 4.33, *SD* = 0.89). 10 out of 12 (91.7%) of the treatment participants who completed the post-treatment feedback questionnaire reported that they felt confident in recommending this programme to a friend.

**Table 4:** Treatment satisfaction with the programme.

**Measure**	**Very poor**		**Poor**		**Fair**		**Good**		**Very good**	
	***N***	**%**	***N***	**%**	***N***	**%**	***N***	**%**	***N***	**%**
Module content	0	0	0	0	2	20	5	50	3	30
Duration of programme	0	0	0	0	5	50	3	30	2	20
Number of face-to-face sessions	0	0	1	10	3	30	2	20	4	40
Length of each face-to-face session (i.e., approximately 50 min)	0	0	0	0	3	30	3	30	4	40
Time intervals in between face-to-face sessions (i.e., meet once every 2 weeks)	0	0	0	0	2	20	5	50	3	30
Homework materials	0	0	0	0	3	30	5	50	2	20
Ease of use for online materials	0	0	0	0	1	10	2	20	7	70

### Qualitative Feedback

Three main themes on improvements to the programme were identified: (1) Therapist contact, (2) module content, and (3) module delivery.

#### Therapist Contact

Several participants cited a desire for more therapist contact for various reasons such as:

a) Having greater assistance in applying the skills taught from the modules, “…having more face to face sessions to support clients so that they can use the skills…effectively, as a result, the program would be more relevant and meet the clients' needs.”;
b) To increase motivation for change, “…I think personally I need to be motivated/reminded/pressured more to do things, e.g., reading the online modules and putting the things into practise.”;
c) Having the space to talk about their conditions and difficulties, “…sometimes, they (patients) are just too embarrassed or stressed out to talk, but maybe by asking them this (what is really bothering them), they are willing to share.”
d) Allowing for customisation of CBT to their issues, “…more sessions with psychologists and some specific CBT techniques to practise”.

#### Module Content

Several participants provided feedback or suggestions for module content:

a) Illness-specific content to include other mental illness, “…more different modules for those suffering from other mental illness such as BPD (Borderline Personality Disorder).and anxiety”.
b) Other patients' testimonies, “…include some videos testimonies or articles of people who have managed to pull through depression. In this way, participants (can) relate and be inspired to take the first step towards self-improvement”.
c) Reducing homework requirements from the modules.
d) Supplementary resources, “…provide more tips and reading recommendations for further read-ups”.·

#### Module Delivery

There was also feedback on module delivery:

a) More visual aids to improve engagement, “…use of graphics is much better than words”; “…by having more visual aid and materials, making the session more interactive”).
b) Increasing the programme duration with longer intervals between face-to-face sessions, to allow for more time to complete the modules and apply the skills learned.
c) Customisable and flexible programme schedule, “…the duration of the whole programme and the time intervals in between face-to-face sessions can be adjusted according to each individual's needs or mental health condition, so that he or she can complete the iCBT programme adequately.”

## Discussion

The present study examined the efficacy and acceptability of a hybrid iCBT programme with three face-to-face sessions for patients presenting with mild to moderate depressive symptoms in Singapore. There were significant reductions in symptoms of depression (moderate to large ESs), anxiety (moderate to large ESs), and psychological distress (moderate ESs) in response to treatment, and no significant reductions in control conditions in both per-protocol and intention-to-treat analyses. However, the between-group analysis between treatment and control groups in the initial comparison arm was only significant for the PHQ-9 (moderate ES) for the intention-to-treat analysis and comparisons between treatment and control responses were not significant.

Examining the means and individual data revealed a few contributors to the lack of significance in the comparison between treatment and controls. The between-group analysis was relatively underpowered as it did not include data from the cross-over arm. In the initial treatment group, the post-treatment follow-up was used as a control condition. However, it appeared that participants continued to improve in their symptoms during the post-treatment period. Finally, the nature of the cross-over analysis meant that participants who dropped out even in the follow-up period were not included in the per-protocol analyses and diluted effects seen in the intention-to-treat analysis. The improvements in symptoms of depression (moderate to large ESs), anxiety (large ESs), and psychological distress (moderate ESs) observed at post-treatment were sustained at follow-up. The ESs are generally consistent with the results from similar iCBT studies (Choi et al., 2012; Titov et al., 2013) and other transdiagnostic face-to-face programs (McEvoy et al., 2009).

A majority of the participants who completed the post-treatment questionnaires were satisfied with the programme. Specifically, 90 percent of participants were willing to recommend it to others, stating they were most pleased with the convenience, teaching skills, and ability to revisit the materials at their convenience. The unfamiliarity with iCBT might partly account for anecdotal reports by some participants in our study, whereby they found face-to-face sessions beneficial and preferred more of such sessions. Talking to a clinician allowed them to express their feelings and receive guidance on applying their learned techniques. This is supported by a previous survey conducted on Chinese-speaking international students in Australia which found that more respondents reported a preference for face-to-face treatments over internet treatments (Lu et al., 2013). As such, a hybrid iCBT model which includes regular face-to-face sessions may be more acceptable for certain Asian populations than solely offering only phone or email support.

The completion rates in our study were slightly lower (60.9 vs. 68%) than a similar study conducted among Chinese participants (Choi et al., 2012). One difference between the two studies is Choi's et al. (2012) study recruited volunteers from the community, whereas participants from our study were patients referred by their clinicians at a tertiary, acute mental health hospital. In addition, in terms of demographics, our sample was younger, had a smaller proportion of married individuals and was less educated than Choi's et al. (2012) participants. It has been shown that younger age, being single and lower education are associated with higher drop-out rates from psychotherapy (Fenger et al., 2011).

In light of the potential benefits of internet treatments and the general acceptability of such treatments amongst this population, the low recruitment rate (i.e., only 66 people were referred over 2 years) in this study is surprising. Given the limited application of internet treatments to Asian populations, depressed individuals in Singapore might be hesitant to participate in iCBT programs due to their unfamiliarity with what such programs entail. This finding is supported by Mitchell and Gordon (2007) study which indicates that people who do not have prior exposure to computer-based CBT, including iCBT, generally do not possess favourable attitudes towards such approaches.

## Limitations

The results of the present study need to be interpreted in light of the limitations of the study, which include a relatively small sample size and high attrition rates in the completion of outcome measures over time. Additionally, the study did not conduct formal diagnostic assessments but relied on the diagnoses provided by the patients' psychiatrists and used thresholds from self-reported measures as a surrogate for clinical remission.

Nonetheless, improvements on the primary and secondary outcome measures occurred in the clinically expected direction and the analyses suggested that our findings could not be explained by biases in participants who dropped out. The treatment group and cross-over arms showed consistency in their ESs in response to treatment, providing further support for the reliability of our findings.

## Conclusion

This current study is the first of its kind to provide preliminary evidence for the efficacy and acceptability of iCBT for depression in Singapore. In light of the ongoing Covid-19 global crisis, iCBT has assumed increasing importance as it can allow for the continual provision of mental health services when access to traditional services are disrupted (Liu et al., 2020). As other programmes have shown wider adoption across the community in other countries (Richards et al., 2015), this study provides valuable information for further development of local internet-based programmes and early groundwork for their broader adoption in Asian cultures.

## Data Availability Statement

The raw data supporting the conclusions of this article will be made available by the authors, without undue reservation, upon request.

## Ethics Statement

The studies involving human participants were reviewed and approved by National Healthcare Group, Domain-Specific Review Board ethics committee (Protocol Record 2015/00404). The patients/participants provided their written informed consent to participate in this study.

## Author Contributions

SL contributed to the design of the trial, recruitment, provision of interventions, and write-up of manuscript. HA contributed to recruitment and provision of interventions. TK contributed to recruitment, data collection and cleaning, write-up of parts of methodology, and preliminary review of manuscript. SN contributed to data cleaning and preliminary review of manuscript. TT contributed to data cleaning and review of manuscript. GT contributed to the statistical analysis and write-up of manuscript.

## Conflict of Interest

The authors declare that the research was conducted in the absence of any commercial or financial relationships that could be construed as a potential conflict of interest.

## Publisher's Note

All claims expressed in this article are solely those of the authors and do not necessarily represent those of their affiliated organizations, or those of the publisher, the editors and the reviewers. Any product that may be evaluated in this article, or claim that may be made by its manufacturer, is not guaranteed or endorsed by the publisher.
