# Supplementary material for: A Randomised Controlled Trial of Clinician-Guided Internet-Based Cognitive Behavioural Therapy for Depressed Patients in Singapore
Source: Front Psychol. 2021 Jul 29;12:668384. doi: 10.3389/fpsyg.2021.668384 (PMC8359863; doi:10.3389/fpsyg.2021.668384)
Supplement: Supplementary file 1 [file Data_Sheet_1.pdf]

Supplementary Material

Supplementary Data

Initial comparison arm

Per-protocol Treatment Response

Per-protocol analysis revealed that in the initial comparison arm, the treatment group had significantly lower post-treatment scores as compared to pre-treatment on the PHQ-9 ($F_{1,15} = 10.80$, $p = 0.01$). There were no significant changes in GAD-7 ($F_{1,15} = 2.48$, $p = 0.14$), WSAS ($F_{1,15} = 1.75$, $p = 0.21$) or K-10 ($F_{1,15} = 0.67$, $p = 0.43$).

Per-protocol Control Response

Per-protocol analysis showed that in the initial comparison arm, there were no significant changes in the control group on the PHQ-9 ($F_{1,7} = 0.26$, $p = 0.63$), GAD-7 ($F_{1,7} = 0.05$, $p = 0.84$), WSAS ($F_{1,7} = 0.004$, $p = 0.95$), and K-10 ($F_{1,7} = 0.20$, $p = 0.67$).

Intention-to-treat Treatment Response

Intention-to-treat analysis analysis revealed that in the initial comparison arm, the treatment group had marginally significantly lower post-treatment scores as compared to pre-treatment on the PHQ-9 ($F_{1,21} = 4.20$, $p = 0.05$). There was no significant change in GAD-7 ($F_{1,21} = 0.30$, $p = 0.59$), WSAS ($F_{1,21} = 2.56$, $p = 0.13$) and K-10 ($F_{1,21} = 0.45$, $p = 0.51$).

Intention-to-treat Control Response

Intention-to-treat analysis showed that in the initial comparison arm, there were no significant changes in the control group on the PHQ-9, ($F_{1,21} = 1.40$, p = 0.25), GAD-7 ($F_{1,21} = 0.01$, p = 0.92), WSAS ($F_{1,21} = 0.04$, $p = 0.85$) and K-10 ($F_{1,21} = 0.13$, p = 0.72).

Cross-over arm

Per-protocol Treatment Response

Per-protocol analysis revealed that in the cross-over arm, the cross-over treatment was associated with significantly lower post-treatment scores as compared to pre-treatment on the GAD-7 ($F_{1,11} = 10.37$, $p = 0.01$) and K-10 ($F_{1,11} = 5.21$, $p = 0.04$). There were no significant changes observed in the PHQ-9 ($F_{1,11} = 1.78$, $p = 0.21$) and the WSAS ($F_{1,11} = 0.57$, $p = 0.47$).

Per-protocol Control Response

Per-protocol analysis revealed in the initial treatment group there were no significant changes with time from post-treatment to follow-up in the PHQ-9 ($F_{1,18} = 0.43$, $p = 0.52$), GAD-7 ($F_{1,18} = 0.64$, $p = 0.44$), WSAS ($F_{1,18} = 0.01$, $p = 0.92$), and K-10 ($F_{1,18} = 0.75$, $p = 0.40$).

Intention-to-treat Treatment Response

Intention-to-treat analysis revealed that in the cross-over arm, the cross-over treatment was associated with significantly lower post-treatment scores as compared to pre-treatment on the GAD-7 ($F_{1,22}$ = 10.37, p =.004) and K-10 ($F_{1,22}$ = 7.78, p = 0.01).There were no significant changes observed in the PHQ-9 ($F_{1,22}$ = 2.79, p = 0.11)and the WSAS ($F_{1,22}$ = 1.38, p = 0.25).

Intention-to-treat Control Response

Per-protocol analysis revealed in the initial treatment group there were no significant changes with time from post-treatment to follow-up in the PHQ-9 ($F_{1,22}$ = 0.63, p = 0.44), GAD-7 ($F_{1,22}$ = 0.37, p = 0.55), WSAS ($F_{1,22}$ = 0.01, p = 0.92) and K-10 ($F_{1,22}$ = 0.66, p = 0.43).

.
